# A Novel *Nobecovirus* in an *Epomophorus wahlbergi* Bat from Nairobi, Kenya

**DOI:** 10.3390/v17040557

**Published:** 2025-04-12

**Authors:** Meredith C. VanAcker, Koray Ergunay, Paul W. Webala, Maureen Kamau, Janerose Mutura, Rashid Lebunge, Griphin Ochieng Ochola, Brian P. Bourke, Emily G. McDermott, Nicole L. Achee, Le Jiang, John P. Grieco, Erick Keter, Audrey Musanga, Suzan Murray, Jared A. Stabach, Meggan E. Craft, Eric M. Fèvre, Yvonne-Marie Linton, James Hassell

**Affiliations:** 1 Department of Evolution, Ecology, and Organismal Biology, University of California, Riverside, CA 92521, USA; 2 Global Health Program, Smithsonian Institution, National Zoo and Conservation Biology Institute, Washington, DC 20008, USA; 3 Walter Reed Biosystematics Unit (WRBU), Smithsonian Institution, Museum Support Center, Suitland, MD 20746, USA; ekoray@hacettepe.edu.tr (K.E.);; 4 One Health Branch, Walter Reed Army Institute of Research (WRAIR), Silver Spring, MD 20910, USA; 5 Department of Entomology, Smithsonian Institution, National Museum of Natural History (NMNH), Washington, DC 20560, USA; 6 Virology Unit, Department of Medical Microbiology, Faculty of Medicine, Hacettepe University, Ankara 06100, Turkey; 7 Department of Forestry and Wildlife Management, Maasai Mara University, Narok 20500, Kenya; pwebala@mmarau.ac.ke; 8 Mpala Research Centre (MRC), Nanyuki 10400, Kenya; maureen.wanjiku.k@gmail.com (M.K.); jrmutura@gmail.com (J.M.); rashbunge@gmail.com (R.L.);; 9 Department of Entomology and Plant Pathology, University of Arkansas, Fayetteville, AR 72701, USA; emcdermo@uark.edu; 10 Department of Biological Sciences, Eck Institute for Global Health, University of Notre Dame, Notre Dame, IN 46556, USA; nachee@nd.edu (N.L.A.);; 11 Viral and Rickettsial Diseases Department, Infectious Diseases Directorate, Naval Medical Research Center (NMRC), 503 Robert Grant Avenue, Silver Spring, MD 20910, USA; 12 Department of Wildlife Management, University of Eldoret, Eldoret 30100, Kenya; erickketer224@gmail.com; 13 College of Agriculture and Veterinary Sciences, University of Nairobi, Nairobi 00100, Kenya; 14 Conservation Ecology Center, Smithsonian National Zoo and Conservation Biology Institute, Front Royal, VA 22630, USA; stabachj@si.edu; 15 Department of Ecology, Evolution and Behavior, College of Biological Sciences, University of Minnesota, St. Paul, MN 55108, USA; craft004@umn.edu; 16 Institute of Infection, Veterinary and Ecological Sciences, University of Liverpool, Liverpool L69 3BX, UK; eric.fevre@liverpool.ac.uk; 17 International Livestock Research Institute (ILRI), Nairobi 00100, Kenya; 18 Department of Epidemiology of Microbial Diseases, Yale School of Public Health, New Haven, CT 06520, USA

**Keywords:** East Africa, urban bat-borne coronavirus, peridomestic habitat, Betacoronavirus, metagenomics, Nobecovirus, wildlife–livestock–human interface, *Eidolon helvum*

## Abstract

Most human emerging infectious diseases are zoonotic, originating in animal hosts prior to spillover to humans. Prioritizing the surveillance of wildlife that overlaps with humans and human activities can increase the likelihood of detecting viruses with a high potential for human infection. Here, we obtained fecal swabs from two fruit bat species—*Eidolon helvum* (*n* = 6) and *Epomophorus wahlbergi* (*n* = 43) (family Pteropodidae)—in peridomestic habitats in Nairobi, Kenya, and used metagenome sequencing to detect microorganisms. A near-complete genome of a novel virus assigned taxonomically to the *Coronaviridae* family *Betacoronavirus* genus and *Nobecovirus* subclade was characterized from *E. wahlbergi*. Phylogenetic analysis indicates this unique *Nobecovirus* clade shares a common ancestor with Eidolon/Rousettus Nobecovirus subclades isolated from Madagascar, Kenya, and Cameroon. Recombination was detected across open reading frames, except the spike protein, in all BOOTSCAN analyses, indicating intra-host coinfection and genetic exchange between genome regions. Although Nobecoviruses are currently bat-specific and are not known to be zoonotic, the propensity of coronaviruses to undergo frequent recombination events and the location of the virus alongside high human and livestock densities in one of East Africa’s most rapidly developing cities justifies continued surveillance of animal viruses in high-risk urban landscapes.

## 1. Introduction

Infectious diseases are a major threat to human, wildlife, and livestock health and are a significant burden on global public health and economic systems. Efforts to elucidate the environmental, epidemiological, and ecological factors that drive zoonotic viral emergence specifically have increased our understanding of the detrimental and complex role of land-use change in spillover [1,2] while exposing the expansive knowledge gap on host–virus associations driven by unknown viral diversity [3]. A recent estimate posits that 10,000 viruses with zoonotic potential circulate in non-human mammals [3], the continued discovery of which greatly improves our understanding of global viral emergence patterns.

Several studies have examined whether taxonomic animal groups have predictable patterns in the number of zoonoses they host [4,5,6]. If certain host groups contribute disproportionately to the zoonotic potential of virus species [4], efforts toward zoonotic discovery and outbreak preparedness and response could be strategically prioritized toward those groups. Bats (Mammalia: Chiroptera) specifically have been hypothesized to be a “special” reservoir host based on immunological mechanisms that allow bats to tolerate viral infection [7,8,9] and due to the links between bat-derived coronaviruses and human epidemics, including severe acute respiratory syndrome (SARS-CoV), Middle East respiratory syndrome-related coronavirus (MERS-CoV) [10], and the ongoing SARS-CoV-2 pandemic—whose ancestral genome relates to bat Sarbecoviruses [11,12]. Alternative hypotheses have been proposed to predict which host species will likely harbor the next human-infecting virus, including an animal order’s evolutionary divergence from humans [4]; the life history strategies of taxonomic groups, such as rodents, that facilitate sympatry with humans [6]; and more recently, that the abundance of human-infecting viruses are linked to the number of viruses maintained by each reservoir group, predicted by a group’s species richness [5,13]. Although these hypotheses are still being debated, they serve as a guide for key factors to consider when conducting viral surveillance of wildlife reservoirs.

Urbanization is a key driver of zoonotic disease emergence, as anthropogenic disturbances from land conversion alter contact between humans, animals, and pathogens and can result in pathogen spillover across novel interfaces. It is critical to monitor the viral diversity circulating in urban reservoir hosts considering the role of urban development in driving zoonotic emergence and because subsequent pathogen spread through human populations in an urban landscape may occur at a higher frequency than in natural and rural landscapes. Bats and rodents frequently share habitats with humans and human-dominated landscapes, such as in cities, and represent the richest mammalian orders globally [14]. Further, the Afrotropical region (Africa south of the Sahara with Madagascar) hosts 31% of mammal diversity across the tropics globally [15], with approximately 20% of all recognized bat species occurring in continental Africa [16]. Over 100 species of bats are found in Kenya alone [17,18]. With diverse bat species occupying habitats that overlap with rapid, often unplanned urbanization, Nairobi, Kenya, is an ideal location for the surveillance of urban bats. Further, novel interactions and microbial exchanges between wildlife reservoirs, livestock, and humans were previously documented in Nairobi, flagging it as a potentially high-risk landscape for pathogen spillover [19]. Prior work has identified a range of viruses in Kenyan bat species, including adenoviruses, astroviruses, caliciviruses, coronaviruses, rhabdoviruses, rotaviruses, paramyxoviruses, and filoviruses [20,21,22,23,24,25,26].

Members of the family *Coronaviridae* are enveloped, positive-sense RNA viruses that infect four of the seven classes of vertebrates: mammals and birds (Orthocoronaviruses), amphibians (Letoviruses), and bony fish (Pitoviruses) [27]. In the subfamily *Orthocoronavirinae*, four genera are recognized, where Alphacoronavirus and Betacoronavirus are primarily associated with bats as hosts. The *Betacoronavirus* genus can be further broken down into subgenera: *Sarbecovirus* (hosted by bats in the family Rhinolophidae), *Merbecovirus* (hosted by bats in the family Vespertilionidae), *Nobecovirus* (hosted by bats in the family Pteropodidae), and *Hibecovirus* (hosted by bats in family Hipposideridae) [28,29,30,31]. Another subgenus, *Embecovirus*, is primarily associated with rodents and bovids, though a few bat hosts have been described. Here, we utilized metagenome sequencing to ensure unbiased pathogen screening of bat-derived samples and to characterize the genome of the novel *Betacoronavirus* we detected in an urban fruit bat. This work provides integral information on the complex epidemiology of coronaviruses in wild animal populations, especially those adapted to anthropogenically dominated landscapes with high densities of humans and livestock.

## 2. Materials and Methods

### 2.1. Study Area and Bat Sampling

**Figure 1:**
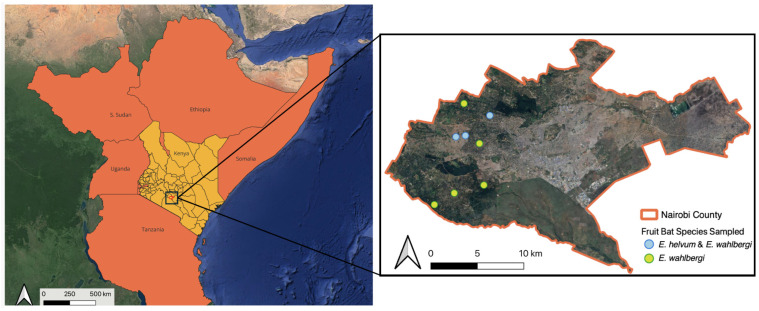
Regional map of East Africa with Nairobi County highlighted. Within Nairobi County (**right**), we trapped foraging fruit bats in west and central Nairobi where suitable peridomestic habitats were found. Blue points indicate households where *E. helvum* and *E. wahlbergi* were trapped and sampled, and green points represent households where only *E. wahlbergi* was trapped and sampled.

Between June and August 2023, bat samples were obtained from *E. wahlbergi* and *E. helvum* at eight households in Nairobi, Kenya (Figure 1). Individual households were selected based on suitable habitats present for fruit bat foraging (e.g., fruiting trees), the presence of flyways (e.g., in riverine vegetation), and recent eco-epidemiological data that indicated the presence of *E. wahlbergi* and *E. helvum* in the neighborhood [32].

Bats were captured using two to three mist nets that were set up before sunset at flyways within the residential property and checked every 15 min. Only *E. wahlbergi* and *E. helvum* were retained for processing, while all other species were released at capture points. One trap night was conducted at each site and took place between the hours of 17:00 and 24:00 or until 9 targeted bats were trapped and sampled. Bats were placed in separate clean cloth bags to prevent cross-contamination and processed in order of capture. During bat handling, individuals were identified with species using published keys [17]. We recorded the body weight and forearm length of each bat and collected oropharyngeal and rectal swabs, whole blood, and fecal and urine samples when available (IACUC #SI-22051 and research permit WRTI-0247-11-22). All samples were directly stored in DNA/RNA Shield (Zymo Research, Irvine, CA, USA) for sample preservation and transferred to a −80 °C freezer within 24–48 h from field collection. Bats were released following sample collection from the processing area near the mist nets where individuals were trapped.

### 2.2. Sample Processing and Library Preparation

Swabs were individually processed for total nucleic acids, as described in detail in the protocol in [33]. Briefly, the samples were lysed in Proteinase K and ATL Lysis Buffer with Reagent DX (Qiagen, Valencia, CA, USA) with 0.1 mm zirconium oxide beads using a Bullet Blender (Next Advance, Troy, NY, USA). The lysate was centrifuged, and the supernatant was extracted using the IndiMag Pathogen Kit (Indical Bioscience, Leipzig, Germany) with the KingFisher™ Flex Purification System (ThermoFisher Scientific, Waltham, MA, USA). Nucleic acid concentrations were measured using a Qubit 4 Fluorometer following manufacturer instructions.

Oxford Nanopore Sequencing (ONS) cDNA libraries were prepared using NEBNext Ultra II RNA First Strand and Non-Directional RNA Second Strand Synthesis modules, utilizing random primer mix (New England Biolabs, Ipswich, MA, USA), following DNase treatment, as previously described [34]. Subsequently, double-stranded cDNA libraries were generated using the NEBNext Ultra II End repair/dA-tailing Module and Quick Ligation Modules (New England Biolabs), as well as a Ligation Sequencing Kit SQK-LSK109 (Oxford Nanopore Technologies, Oxford, UK). Cleanup and quantification were carried out using Agencourt AMPure XP reagent (Beckman Coulter Biosciences, Indianapolis, IN, USA) and a Qubit dsDNA HS Assay Kit (ThermoFisher Scientific). Samples were individually barcoded with the Native Barcoding Expansion 96—EXP-NBD 196 (Oxford Nanopore Technologies) and combined for sequencing. Sequencing libraries containing 24 barcoded pools were loaded on a single ONT R9.4.1 flow cell and run on a MinION Mk1C device (Oxford Nanopore Technologies) for 48 h.

### 2.3. Data Processing and Phylogenetic Analysis

Base calling and demultiplexing were accomplished on the device with MinKNOW operating software v21.11.7 (Oxford Nanopore Technologies) and Guppy v5.1.13 [35]. Raw reads were trimmed with Porechop to remove adapter sequences and then filtered with NanoFilt to remove reads with q-scores ≤9 and read lengths ≤100 bp [35,36]. Bat genomes were further removed using Minimap2 v2.24 and Samtools v1.9 [37,38]. The processed data were aligned to the National Center for Biotechnology Information (NCBI) non-redundant (NR) database using DIAMOND v2.0.14 and visualized using MEGAN6 (v6.25.9) [39,40]. The Flye assembler, developed for long single-molecule sequencing reads, was used for de novo assemblies [41]. Sequences were handled using Geneious Prime (v.2024.0.4) (Biomatters Ltd., Auckland, New Zealand). The BLASTn (2.13.0) and BLASTp (2.13.0) algorithms were used for similarity searches in the NCBI database [42]. CLUSTALW (2.0.11) was used for sequence alignment and pairwise comparisons [43]. Protein domain and motif searches were performed using the NCBI conserved domain search tool and MOTIF Search in the PFAM database [44,45].

Phylogenetic analysis was performed on sequences using IQ-TREE 2 and MEGA [46,47]. In IQ-TREE, the optimal evolutionary models and partitioning schemes were determined for amino acid sequence alignment using the automatic model selection tools (-mMFP+MERGE). Amino acid models were restricted to those designed for viral sequences (-msub viral). A 70% majority-rule consensus tree was constructed by maximum likelihood using 1000 replicates from the ultrafast bootstrap approximation approach (UFBoot) [48]. The UFBoot support values are more unbiased than normal bootstrap support, and significant clade support is considered at ≥95% [48,49]. Standard bootstrap analysis was carried out using MEGA v11.0.13 [47] for 500 replicates. The optimal analysis models were selected using the built-in “Find Best DNA/protein-substitution model” tools. Maximum likelihood trees based on nucleotide sequences were constructed using the General Time Reversible (GTR) model with a discrete Gamma distribution (+G) (ORF1a) and the General Time Reversible (GTR) model with a discrete Gamma distribution and invariant sites (+G+I) (ORF1b). Potential genetic exchange and recombination events were assessed using the RDP, GENECONV, BOOTSCAN, MAXCHI, CHIMAERA, SISCAN, and 3SEQ tools in automated and manual analyses with default settings [50]. BOOTSCAN plots were generated using SimPlot (version 3.5.1) [51].

## 3. Results

A total of 49 rectal swabs were processed from two fruit bat species, Wahlberg’s epauletted fruit bats (*E. wahlbergi*, 43/49, 87.7%) and straw-colored fruit bats (*E. helvum*, 6/49, 12.2%). Viral contigs were generated by de novo assembly from a single *E. wahlbergi* sample (1/49, 2.0%). Through the remapping of reads to assembled contigs and producing alignments, a single virus contig was generated that revealed the identities of several Nobecoviruses from Africa in BLAST queries.

### 3.1. Genome Annotation and Phylogenetic and Recombination Analyses

The virus contig (referred to as NRB24) comprised 21,973 nucleotides, covering 75–76% of bat Nobecovirus genomes. Seven open reading frames (ORFs) were identified, including partial ORF1a (1−7099) and complete ORF1b (7099–15,114), spike (S) glycoprotein (15,086−18,910), ORF3 (18,911–19,609), envelope (E) protein (19,609−19,836), membrane (M) glycoprotein (19,841−20,509), and nucleocapsid (N) protein (20,564−21,973, Figure S1). As observed in coronaviruses, ORF1a and ORF1b partially overlap in NRB24, where ORF1b is in the −1 reading frame relative to the ORF1a stop codon (7097−7099), enabling the expression of ORF1b-encoded protein expression by cis-acting RNA elements that direct a fraction of elongating ribosomes to slip (programmed ribosomal frameshifting) [52].

**Figure 2:**
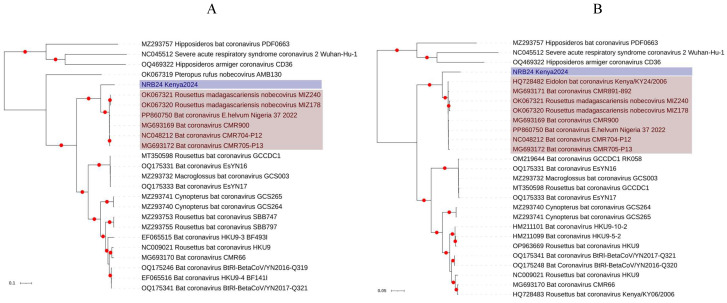
The maximum likelihood consensus tree of coronavirus ORF1a ((**A**): 2419 amino acids) and ORF1b ((**B**): 2528 amino acids) sequences. The trees were constructed using 1000 replicates. Branches achieving ≥95% bootstrap support are annotated with red dots. Viruses are indicated by GenBank accession, name, and isolate identifier. NRB24 and related virus genomes are marked. SARS-CoV-2 Wuhan-Hu-1 (Sarbecovirus) and *Hipposideros* coronaviruses CD36/PDF0663 (Hibecovirus) were included as outgroups as needed.

**Figure 3:**
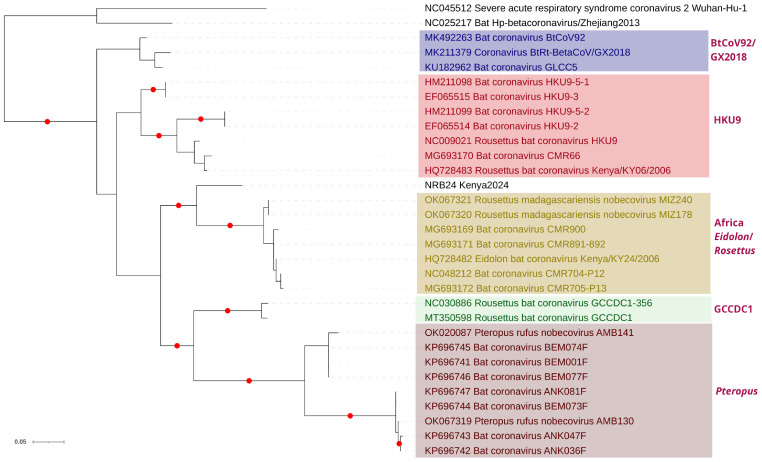
The maximum likelihood consensus tree of the coronavirus RNA-dependent RNA polymerase (RdRp) sequences (256 nucleotides). The trees were constructed using 1000 replicates. Branches achieving ≥95% bootstrap support are annotated with red dots. Viruses are indicated by GenBank accession, name, and isolate identifier. Previously described Nobecovirus clades [53] are marked. SARS-CoV-2 Wuhan-Hu-1 (Sarbecovirus) and bat coronavirus Zhejiang2013 (Hibecovirus) were included as outgroups.

Pairwise comparisons of individual ORFs with Nobecoviruses from Africa revealed identities of up to 77.6% and 91.9% in nucleotide and putative amino acid sequences, respectively (Table S1). Maximum likelihood analysis based on complete putative ORF1a and ORF1b nucleotide and amino acid sequences showed that NRB24 formed a separate subclade, distinct from previously described Nobecovirus clades, with strong bootstrap support (Figure 2 and Figure S2) [53]. Similar tree topologies were observed in maximum likelihood trees constructed using S, ORF3, and N putative amino acid alignments as well (Figure S3). In each tree, NRB24 remained distinct, sharing a common ancestor with the African Nobecovirus clade, which includes all geographically related virus genomes. Moreover, a comparable tree topology was observed during the analysis of a shorter nucleotide fragment encoding for virus RNA-dependent RNA polymerase (RdRp) (Figure 3), which was documented to delineate major Nobecovirus clades [53]. However, the separate grouping of NRB24 was not apparent in the maximum likelihood trees based on putative E and M amino acid alignments, presumably due to their relatively limited sizes (Figure S3).

**Figure 4:**
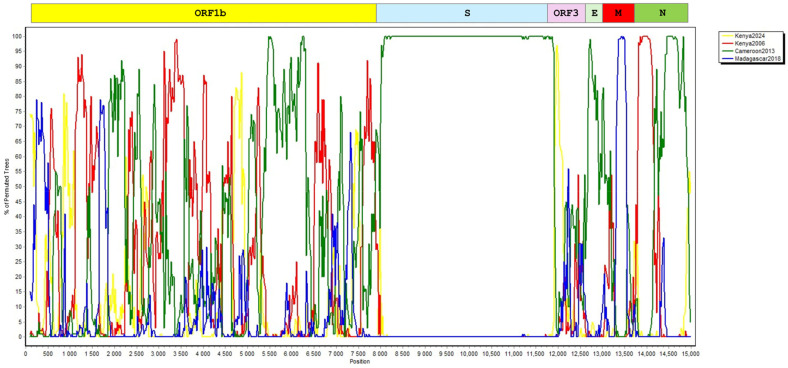
BOOTSCAN plot of African bat Nobecovirus genomes. The alignment encompasses 15096 positions with the corresponding ORFs plotted on top. The plot was prepared within a sliding window of 200 bp, wide-centered on the position plotted, with a 20 bp step size, for 1000 replications (GapStrip: on, neighbor-joining, T/t: 2.0), with the bat coronavirus CMR704-P12 genome (NC048212, *Eidolon helvum*, Cameroon) as the query. Refer to Table 1 for a description of genomes in groups labeled Kenya2024 (NRB24), Kenya2006, Madagascar2018, and Cameroon2013.

We carried out further in silico analyses of complete ORF sequences to detect potential recombinations in Nobecoviruses from Africa, including NRB24. Probable recombinations involving multiple spots were detected using all tools. In the BOOTSCAN plot, recombination signals were observed to involve all ORFs but the S protein (Figure 4), suggesting coinfections and intra-host genetic exchange driving genome diversification.

### 3.2. Analysis of Putative Viral Proteins

We examined the putative amino acid sequences of ORF1a (2365), ORF1b (2579), S glycoprotein (1274), ORF3 (232), E protein (75), M glycoprotein (222), and N protein (469). The ORF1a protein was partially identified with the virus non-structural protein (Nsp), and 3–10 conserved domains were identified (Table S2). Similarly, the complete ORF1b revealed conserved motifs of the RdRp catalytic core, as well as Nsp13–16, with various functions required for virus replication. The domains of the main coronavirus structural proteins, S, E, M, and N were further observed in the NRB24 contig (Table 1).

**Figure 5:**
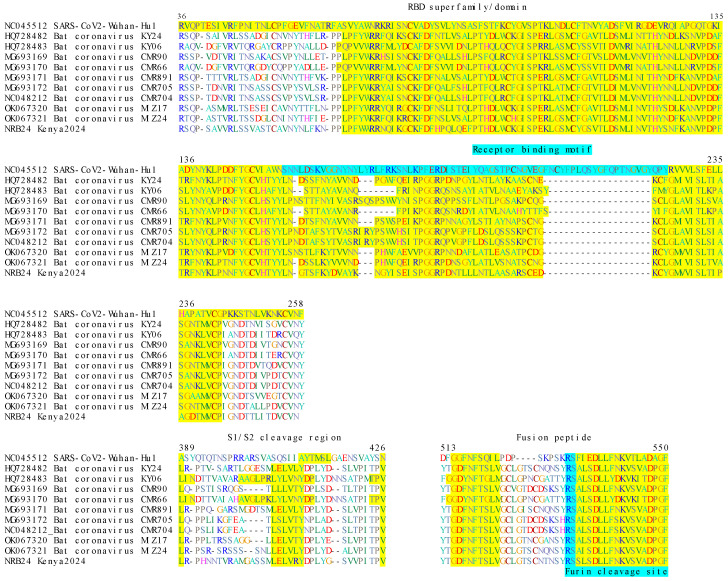
Alignment of domains involved in receptor binding of coronavirus S (spike) protein. Amino acid positions are provided according to the SARS-CoV2 Wuhan-Hu-1 strain. Conserved residues are highlighted (RBD domain, S1/S2 cleavage site, and fusion peptide: yellow; receptor binding motif and furin cleavage site: cyan).

In coronaviruses, the S protein is critical for host range and virulence, as it mediates target cell attachment and the membrane fusion required for virus entry [27]. We identified the S1-S2 subunits, the S1/S2 and furin cleavage sites, the fusion peptide region, and associated motifs in NRB24 (Figure 5). Like other Nobecoviruses, NRB24 lacked the SARS-CoV-2 receptor binding motif and shared S1/S2 and furin cleavage sites and fusion peptide markers with Nobecoviruses.

## 4. Discussion

Here, we report a partial Betacoronavirus genome representing a distinct Nobecovirus subclade related to African Nobecoviruses, detected in a rectal swab from a Wahlberg’s epauletted fruit bat (*E. wahlbergi*) from Nairobi, Kenya. Tentatively named NRB24, the partial viral contig—covering >75% of Nobecovirus genomes—was generated by untargeted metagenome screening. The contig encompassed viral structural proteins (S, E, M, and N), as well as regions encoding for the core replication enzymes and main co-factors encoded by ORF1a and ORF1b. In phylogenetic analyses, it emerged as a novel Nobecovirus subclade, sharing a common ancestor with the Eidolon/Rousettus Nobecovirus subclade from Africa. We detected several functional domains on the NRB24 putative structural and non-structural proteins. Further analysis of the S protein revealed shared markers and cleavage sites with Nobecoviruses. We did not detect RNA derived from NRB24 in oral swabs from the same individual bat, suggesting probable tropism for gastrointestinal tissue.

Zoonotic capacity in particular subgenera, such as Sarbecoviruses (including SARS-CoV and SARS-CoV-2) and Merbecoviruses (including MERS-CoV) has been documented and caused spillover events with a significant health impact [28,29]. So far, no zoonotic potential has been recognized in Nobecoviruses, which are described exclusively in fruit bats of Pteropodidae [27,31]. Moreover, scarce information is available on Nobecovirus cell receptors and target cells. Compared to other bat-associated Sarbecovirus and Merbecoviruses, Nobecoviruses have been observed to infect fewer bat host species, which might indicate host specificity toward *Rousettus* and *Eonycteris* fruit bats (family Pteropodidae) [31,53]. Importantly, our findings describe another Pteropodidae species that can harbor Nobecoviruses.

All Pteropodidae species are distributed in tropical and subtropical areas of the Old World and have been documented to harbor zoonotic viruses, including the Ebola, Marburg, Hendra, and Nipah viruses [31,54]. Despite a lack of direct evidence of Nobecoviruses, examples of virus spillover to humans and recombination events have been documented [55,56], highlighting the potential of viruses hosted by Pteropodidae species. In this study, we documented potential recombination events among various Nobecoviruses of African origin, including the newly described NRB24, despite lacking the S protein, which is crucial for the host range. As exemplified by SARS-CoV and MERS-CoV, recombinations facilitate direct bat-to-human spillover and cross-species emergence via intermediary bridge hosts [28,29,57]. The potential of Nobecovirus to gain zoonotic potential through recombinations should not be underestimated, particularly when fruit bat hosts share habitats with potential intermediate hosts like livestock, as with our sites. It is imperative to continue active surveillance of fruit bat species in the Afrotropics and tropical and subtropical regions of Southeast Asia for emerging corona- and other viruses and to better understand their diverse virome. Despite challenges in identifying adequate bat habitats on private properties in a city environment, limiting our sample size, this study provided novel viral sequences obtained in a non-invasive manner from urban bats, contributing to our knowledge of Nobecoviruses and their bat host associations.

## Figures and Tables

**Table 1:** Comparative topology and conserved domains identified in complete regions in NRB24 and closely related virus genomes.

Host		*Epomophorus wahlbergi*	*Eidolon* *helvum*	*Rousettus* *aegyptiacus*	*Eidolon helvum*					*Rousettus madagascariensis*	
Location, Year		Kenya, 2024	Kenya 2006	Kenya 2006	Cameroon, 2013					Madagascar, 2018	
Isolate		NRB24	KY24	KY06	CMR900	CMR66	CMR891-892	CMR705-P13	CMR704-P12	MIZ178	MIZ240
GenBank Accession		PQ179293	HQ728482.1	HQ728483.1	MG693169.1	MG693170.1	MG693171.1	MG693172.1	NC_048212.1	OK067320.1	OK067321.1
ORF1b	Size	2579	2579	2588	2579	2588	2579	2579	2579	2579	2579
	Catalytic core—RNA polymerase (477363)	1–823	1–823	1–823	1–823	1–823	1–823	1–823	1–823	1–823	1–823
	Nsp13—zinc-binding domain (cl41714)	824–918	824–918	824–918	824–918	824–918	824–918	824–918	824–918	824–918	824–918
	Nsp13—stalk (cl41715)	922–969	922–969	922–969	922–969	922–969	922–969	922–969	922–969	922–969	922–969
	Nsp13—1B domain (cl41715)	973–1051	973–1051	973–1051	973–1051	973–1051	973–1051	973–1051	973–1051	973–1051	973–1051
	Nsp13—helicase domain (cl41748)	1074–1413	1074–1413	1074–1413	1074–1413	1074–1413	1074–1413	1074–1413	1074–1413	1074–1413	1074–1413
	Nsp14 (cl40464)	1428–1950	1428–1951	1428–1952	1428–1951	1428–1952	1428–1951	1428–1951	1428–1951	1428–1951	1428–1951
	Nsp15—N terminal domain (cl40469)	1954–2014	1954–2014	1955–2015	1954–2014	1955–2015	1954–2014	1954–2014	1954–2014	1954–2014	1954–2014
	Nsp15—M domain (cl41717)	2018–2146	2018–2146	2019–2143	2018–2146	2019–2143	2018–2146	2018–2146	2018–2146	2018–2146	2018–2146
	Nsp15—endoribonuclease domain (cl41718)	2144–2292	2144–2292	2141–2289	2144–2292	2141–2289	2144–2292	2144–2292	2144–2292	2144–2292	2144–2292
	Nsp16—methyltransferase (461919, cl41719)	2297–2578	2323–2537	2297–2578	2323–2537	2297–2578	2323–2537	2323–2537	2323–2537	2297–2578	2323–2537
S	Size	1274	1264	1278	1273	1278	1271	1269	1269	1256	1265
	S1—N terminal domain (cd21627)	30–323	33–318	36–327	33–326	36–327	33–325	31–322	31–322	34–312	42–321
	Receptor binding domain (cl09656)	367–521	362–516	371–521	370–528	371–521	369–523	366–524	366–524	356–511	365–519
	S1/S2 cleavage + S2 fusion domain (cd22381)	538–1268	533–1258	538–1273	545–1267	538–1272	540–1265	541–1263	541–1263	528–1250	536–1259
ORF3	Size	232	238	220	238	220	238	239	239	238	238
E	Size	75	75	75	75	75	75	75	75	75	75
	Envelope protein (cl40474)	-	-	-	-	-	-	4–63	4–63	4–63	4–63
M	Size	222	221	222	221	223	221	221	221	221	221
	Matrix protein (cl40475)	11–222	5–222	7–222	8–221	7–223	5–221	10–221	10–221	8–221	8–221
N	Size	469	467	468	468	468	467	470	470	467	467
	Nucleocapsid protein (cl47612)	53–396	53–374	54–375	54–375	54–395	54–375	54–375	54–375	53–374	53–374

Locations are provided according to the individual viral proteins. NCBI accessions are provided in parentheses.

## Data Availability

Raw sequence data generated in this study are available in the NCBI Sequence Read Archive (SRA) under project PRJNA1237873 and the assembled virus genome sequence is available in GenBank under accession number PQ179293.

## Supplementary Materials

The following supporting information can be downloaded at https://www.mdpi.com/article/10.3390/v17040557/s1: Figure S1: NRB24 genome annotation of seven open reading frames and genome coverage. Figure S2: Maximum likelihood tree of coronaviruses ORF1a (7472 nucleotides) and ORF1b (8033 nucleotides). Figure S3: Maximum likelihood consensus tree of coronavirus S (3949 amino acids), E (75 amino acids), ORF3 (266 amino acids), M (215 amino acids), and N (477 amino acids) sequences. Table S1: Pairwise comparison of nucleotide and deduced amino acid sequences of NRB24 and related Nobecovirus ORFs. Table S2: Conserved domains identified in NRB24 partial ORF1a putative amino acid sequence (*n* = 2365).
