# Supplementary material for: A Novel Nobecovirus in an Epomophorus wahlbergi Bat from Nairobi, Kenya
Source: Viruses. 2025 Apr 12;17(4):557. doi: 10.3390/v17040557 (PMC12031378; doi:10.3390/v17040557)
Supplement: Supplementary file 1 [file viruses-17-00557-s001.zip › viruses-3485483-supplementary.pdf]

Supplementary Materials

VanAcker et al., 2025. A novel *Nobecovirus* in an *Epomophorus wahlbergi* bat from Nairobi, Kenya

Figure S1: NRB24 genome annotation of seven open reading frames and genome coverage.

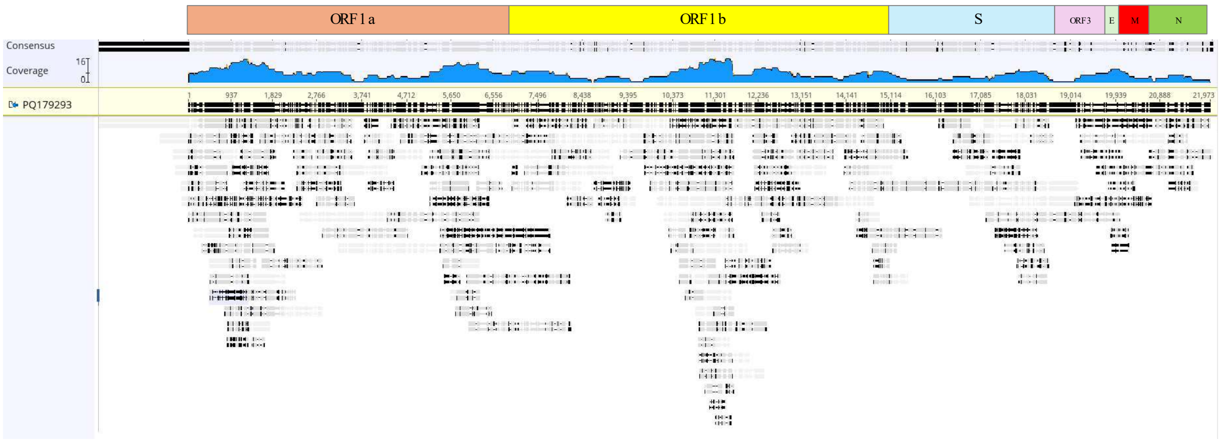

Figure S2: The maximum likelihood tree of the coronavirus ORF1a (7472 nucleotides) and ORF1b (8033 nucleotides).

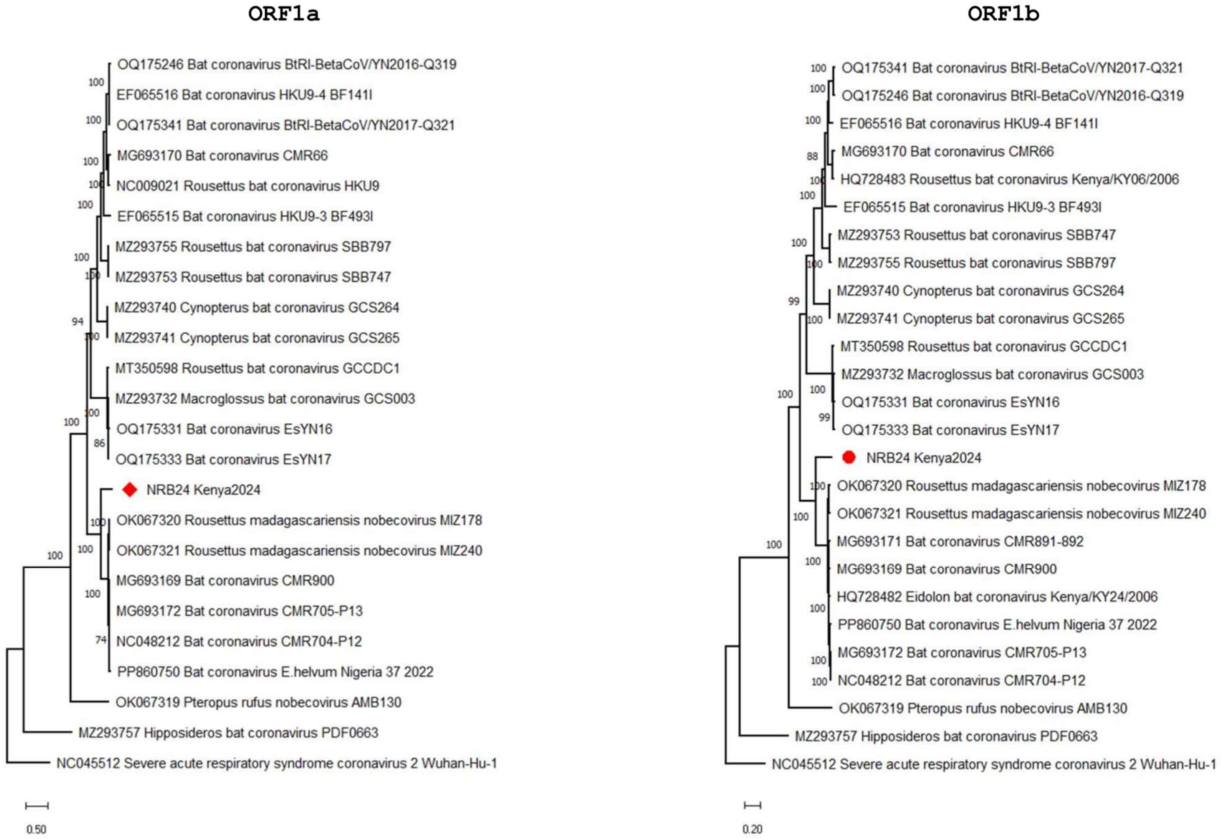

Figure S3: The maximum likelihood consensus tree of the coronavirus S (3949 amino acids), E (75 amino acids), ORF3 (266 amino acids), M (215 amino acids) and N (477 amino acids) sequences. The trees are constructed using 1000 replicates. Branches achieving ≥95% bootstrap support are annotated with red dots. Viruses are indicated by GenBank accession, name and isolate identifier. NRB24 and related virus genomes are marked. SARS-CoV-2 Wuhan-Hu-1 (Sarbecovirus) and *Hipposideros* coronaviruses CD36/ PDF0663 (Hibecovirus) were included as outgroups as needed.

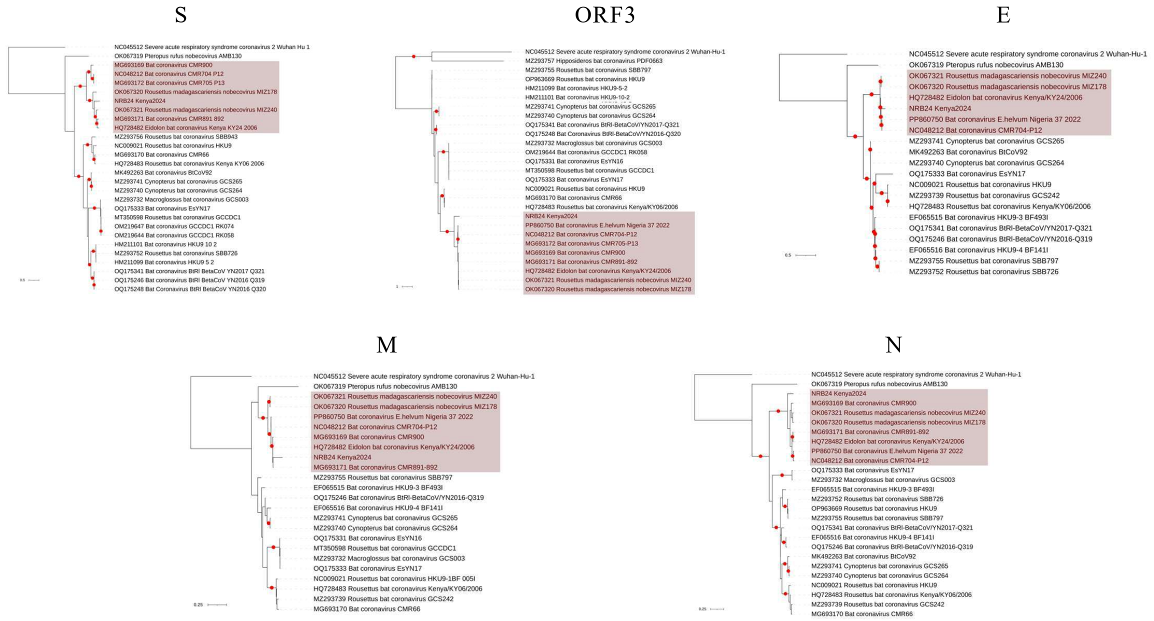

Table S1: Pairwise comparison of nucleotide and deduced amino acid sequences of NRB24 and related nobecovirus ORFs.

ORF1a	Nucleotide (n=7555)										Amino acid (n=2689)									
	HQ728 482	HQ728 483	MG693 169	MG693 170	MG693 171	MG69 3172	NC04 8212	OK06 7320	OK06732 1	NRB24	HQ728 482	HQ728 483	MG693 169	MG693 170	MG693 171	MG693 172	NC048 212	OK067 320	OK067 321	NRB24
HQ728482. Eidolon bat coronavirus/Kenya/KY24/2006	ID	0.746	0.408	0.744	0.409	0.411	0.41	0.406	0.407	0.387	ID	0.828	0.108	0.109	0.109	0.109	0.108	0.109	0.109	0.109
HQ728483. Rousettus bat coronavirus/Kenya/KY06/2006	0.746	ID	0.4	0.971	0.401	0.402	0.401	0.399	0.399	0.385	0.828	ID	0.109	0.104	0.109	0.109	0.109	0.11	0.11	0.109
MG693169. Bat coronavirus CMR900	0.408	0.4	ID	0.398	0.986	0.982	0.982	0.961	0.962	0.776	0.108	0.109	ID	0.691	0.994	0.994	0.996	0.983	0.985	0.847
MG693170. Bat coronavirus CMR66	0.744	0.971	0.398	ID	0.4	0.4	0.399	0.397	0.397	0.382	0.109	0.104	0.691	ID	0.691	0.691	0.692	0.692	0.691	0.678
MG693171. Bat coronavirus CMR891-892	0.409	0.401	0.986	0.4	ID	0.983	0.984	0.961	0.962	0.774	0.109	0.109	0.994	0.691	ID	0.994	0.994	0.982	0.984	0.846
MG693172. Bat coronavirus CMR705-P13	0.411	0.402	0.982	0.4	0.983	ID	0.998	0.962	0.962	0.775	0.109	0.109	0.994	0.691	0.994	ID	0.997	0.982	0.984	0.846
NC048212. Bat coronavirus CMR704-P12	0.41	0.401	0.982	0.399	0.984	0.998	ID	0.963	0.963	0.775	0.108	0.109	0.996	0.692	0.994	0.997	ID	0.983	0.986	0.847
OK067320. Rousettus madagascariensis nobecovirus MIZ178	0.406	0.399	0.961	0.397	0.961	0.962	0.963	ID	0.996	0.775	0.109	0.11	0.983	0.692	0.982	0.982	0.983	ID	0.997	0.84
OK067321. Rousettus madagascariensis nobecovirus MIZ240	0.407	0.399	0.962	0.397	0.962	0.962	0.963	0.996	ID	0.776	0.109	0.11	0.985	0.691	0.984	0.984	0.986	0.997	ID	0.842
NRB24	0.387	0.385	0.776	0.382	0.774	0.775	0.775	0.775	0.776	ID	0.109	0.109	0.847	0.678	0.846	0.846	0.847	0.84	0.842	ID

ORF1b	Nucleotide (n=7743)										Amino acid (n=2509)									
	HQ728 482	HQ728 483	MG693 169	MG693 170	MG693 171	MG693 172	NC048 212	OK067 320	OK067 321	NRB24	HQ728 482	HQ728 483	MG693 169	MG693 170	MG693 171	MG693 172	NC048 212	OK067 320	OK067 321	NRB24
HQ728482. Eidolon bat coronavirus/Kenya/KY24/2006	ID	0.744	0.979	0.743	0.975	0.976	0.978	0.959	0.961	0.532	ID	0.83	0.995	0.829	0.995	0.992	0.996	0.991	0.991	0.919
HQ728483. Rousettus bat coronavirus/Kenya/KY06/2006	0.744	ID	0.745	0.971	0.746	0.746	0.748	0.744	0.744	0.489	0.83	ID	0.829	0.993	0.83	0.827	0.831	0.829	0.826	0.811
MG693169. Bat coronavirus CMR900	0.979	0.745	ID	0.743	0.974	0.972	0.973	0.959	0.963	0.533	0.995	0.829	ID	0.829	0.994	0.993	0.996	0.99	0.99	0.919
MG693170. Bat coronavirus CMR66	0.743	0.971	0.743	ID	0.745	0.745	0.746	0.741	0.742	0.489	0.829	0.993	0.829	ID	0.83	0.827	0.83	0.828	0.826	0.812
MG693171. Bat coronavirus CMR891-892	0.975	0.746	0.974	0.745	ID	0.972	0.974	0.957	0.961	0.532	0.995	0.83	0.994	0.83	ID	0.992	0.995	0.99	0.991	0.919
MG693172. Bat coronavirus CMR705-P13	0.976	0.746	0.972	0.745	0.972	ID	0.996	0.954	0.958	0.531	0.992	0.827	0.993	0.827	0.992	ID	0.995	0.988	0.988	0.916
NC048212. Bat coronavirus CMR704-P12	0.978	0.748	0.973	0.746	0.974	0.996	ID	0.955	0.96	0.531	0.996	0.831	0.996	0.83	0.995	0.995	ID	0.991	0.991	0.919
OK067320. Rousettus madagascariensis nobecovirus MIZ178	0.959	0.744	0.959	0.741	0.957	0.954	0.955	ID	0.98	0.534	0.991	0.829	0.99	0.828	0.99	0.988	0.991	ID	0.994	0.916
OK067321. Rousettus madagascariensis nobecovirus MIZ240	0.961	0.744	0.963	0.742	0.961	0.958	0.96	0.98	ID	0.534	0.991	0.826	0.99	0.826	0.991	0.988	0.991	0.994	ID	0.917
NRB24	0.532	0.489	0.533	0.489	0.532	0.531	0.531	0.534	0.534	ID	0.919	0.811	0.919	0.812	0.919	0.916	0.919	0.916	0.917	ID

S	Nucleotide (n=3907)										Amino acid (n=1303)									
	HQ728 482	HQ728 483	MG693 169	MG693 170	MG693 171	MG693 172	NC048 212	OK067 320	OK067 321	NRB24	HQ728 482	HQ728 483	MG693 169	MG693 170	MG693 171	MG693 172	NC048 212	OK067 320	OK067 321	NRB24
HQ728482. Eidolon bat coronavirus/Kenya/KY24/2006	ID	0.744	0.979	0.743	0.975	0.976	0.978	0.959	0.961	0.532	ID	0.83	0.995	0.829	0.995	0.992	0.996	0.991	0.991	0.919
HQ728483. Rousettus bat coronavirus/Kenya/KY06/2006	0.744	ID	0.745	0.971	0.746	0.746	0.748	0.744	0.744	0.489	0.83	ID	0.829	0.993	0.83	0.827	0.831	0.829	0.826	0.811
MG693169. Bat coronavirus CMR900	0.979	0.745	ID	0.743	0.974	0.972	0.973	0.959	0.963	0.533	0.995	0.829	ID	0.829	0.994	0.993	0.996	0.99	0.99	0.919

MG693170. Bat coronavirus CMR66	0.743	0.971	0.743	ID	0.745	0.745	0.746	0.741	0.742	0.489	0.829	0.993	0.829	ID	0.83	0.827	0.83	0.828	0.826	0.812
MG693171. Bat coronavirus CMR891-892	0.975	0.746	0.974	0.745	ID	0.972	0.974	0.957	0.961	0.532	0.995	0.83	0.994	0.83	ID	0.992	0.995	0.99	0.991	0.919
MG693172. Bat coronavirus CMR705-P13	0.976	0.746	0.972	0.745	0.972	ID	0.996	0.954	0.958	0.531	0.992	0.827	0.993	0.827	0.992	ID	0.995	0.988	0.988	0.916
NC048212. Bat coronavirus CMR704-P12	0.978	0.748	0.973	0.746	0.974	0.996	ID	0.955	0.96	0.531	0.996	0.831	0.996	0.83	0.995	0.995	ID	0.991	0.991	0.919
OK067320. Rousettus madagascariensis nobecovirus MIZ178	0.959	0.744	0.959	0.741	0.957	0.954	0.955	ID	0.98	0.534	0.991	0.829	0.99	0.828	0.99	0.988	0.991	ID	0.994	0.916
OK067321. Rousettus madagascariensis nobecovirus MIZ240	0.961	0.744	0.963	0.742	0.961	0.958	0.96	0.98	ID	0.534	0.991	0.826	0.99	0.826	0.991	0.988	0.991	0.994	ID	0.917
NRB24	0.532	0.489	0.533	0.489	0.532	0.531	0.531	0.534	0.534	ID	0.919	0.811	0.919	0.812	0.919	0.916	0.919	0.916	0.917	ID

**ORF3**	Nucleotide (n=675)										Amino acid (n=238)									
	HQ728 482	HQ728 483	MG693 169	MG693 170	MG693 171	MG693 172	NC048 212	OK067 320	OK067 321	NRB24	HQ728 482	HQ728 483	MG693 169	MG693 170	MG693 171	MG693 172	NC048 212	OK067 320	OK067 321	NRB24
HQ728482. Eidolon bat coronavirus/Kenya/KY24/2006	ID	0.535	0.94	0.544	0.968	0.922	0.922	0.926	0.934	0.376	ID	0.373	0.97	0.369	0.987	0.927	0.927	0.944	0.949	0.593
HQ728483. Rousettus bat coronavirus/Kenya/KY06/2006	0.535	ID	0.538	0.959	0.541	0.529	0.529	0.534	0.541	0.26	0.373	ID	0.373	0.972	0.378	0.382	0.382	0.365	0.369	0.388
MG693169. Bat coronavirus CMR900	0.94	0.538	ID	0.54	0.938	0.916	0.916	0.901	0.908	0.37	0.97	0.373	ID	0.369	0.983	0.919	0.919	0.936	0.936	0.601
MG693170. Bat coronavirus CMR66	0.544	0.959	0.54	ID	0.547	0.531	0.531	0.541	0.548	0.262	0.369	0.972	0.369	ID	0.373	0.378	0.378	0.365	0.365	0.384
MG693171. Bat coronavirus CMR891-892	0.968	0.541	0.938	0.547	ID	0.926	0.926	0.934	0.941	0.384	0.987	0.378	0.983	0.373	ID	0.927	0.927	0.949	0.953	0.597
MG693172. Bat coronavirus CMR705-P13	0.922	0.529	0.916	0.531	0.926	ID	1	0.902	0.902	0.373	0.927	0.382	0.919	0.378	0.927	ID	1	0.894	0.898	0.601
NC048212. Bat coronavirus CMR704-P12	0.922	0.529	0.916	0.531	0.926	1	ID	0.902	0.902	0.373	0.927	0.382	0.919	0.378	0.927	1	ID	0.894	0.898	0.601
OK067320. Rousettus madagascariensis nobecovirus MIZ178	0.926	0.534	0.901	0.541	0.934	0.902	0.902	ID	0.97	0.375	0.944	0.365	0.936	0.365	0.949	0.894	0.894	ID	0.949	0.584
OK067321. Rousettus madagascariensis nobecovirus MIZ240	0.934	0.541	0.908	0.548	0.941	0.902	0.902	0.97	ID	0.378	0.949	0.369	0.936	0.365	0.953	0.898	0.898	0.949	ID	0.593
NRB24	0.376	0.26	0.37	0.262	0.384	0.373	0.373	0.375	0.378	ID	0.593	0.388	0.601	0.384	0.597	0.601	0.601	0.584	0.593	ID

**E**	Nucleotide (n=228)										Amino acid (n=75)									
	HQ728 482	HQ728 483	MG693 169	MG693 170	MG693 171	MG693 172	NC048 212	OK067 320	OK067 321	NRB24	HQ728 482	HQ728 483	MG693 169	MG693 170	MG693 171	MG693 172	NC048 212	OK067 320	OK067 321	NRB24
HQ728482. Eidolon bat coronavirus/Kenya/KY24/2006	ID	0.671	0.942	0.662	0.982	0.938	0.938	0.912	0.907	0.473	ID	0.573	0.986	0.573	1	0.973	0.973	0.946	0.946	0.893
HQ728483. Rousettus bat coronavirus/Kenya/KY06/2006	0.671	ID	0.666	0.973	0.675	0.657	0.657	0.657	0.662	0.35	0.573	ID	0.56	0.986	0.573	0.56	0.56	0.546	0.546	0.533
MG693169. Bat coronavirus CMR900	0.942	0.666	ID	0.657	0.96	0.934	0.934	0.899	0.894	0.464	0.986	0.56	ID	0.56	0.986	0.96	0.96	0.933	0.933	0.88
MG693170. Bat coronavirus CMR66	0.662	0.973	0.657	ID	0.666	0.649	0.649	0.657	0.662	0.35	0.573	0.986	0.56	ID	0.573	0.56	0.56	0.546	0.546	0.533
MG693171. Bat coronavirus CMR891-892	0.982	0.675	0.96	0.666	ID	0.947	0.947	0.903	0.899	0.464	1	0.573	0.986	0.573	ID	0.973	0.973	0.946	0.946	0.893
MG693172. Bat coronavirus CMR705-P13	0.938	0.657	0.934	0.649	0.947	ID	1	0.872	0.868	0.464	0.973	0.56	0.96	0.56	0.973	ID	1	0.92	0.92	0.893
NC048212. Bat coronavirus CMR704-P12	0.938	0.657	0.934	0.649	0.947	1	ID	0.872	0.868	0.464	0.973	0.56	0.96	0.56	0.973	1	ID	0.92	0.92	0.893
OK067320. Rousettus madagascariensis nobecovirus MIZ178	0.912	0.657	0.899	0.657	0.903	0.872	0.872	ID	0.995	0.469	0.946	0.546	0.933	0.546	0.946	0.92	0.92	ID	1	0.866
OK067321. Rousettus madagascariensis nobecovirus MIZ240	0.907	0.662	0.894	0.662	0.899	0.868	0.868	0.995	ID	0.473	0.946	0.546	0.933	0.546	0.946	0.92	0.92	1	ID	0.866

NRB24	0.473	0.35	0.464	0.35	0.464	0.464	0.464	0.469	0.473	ID	0.893	0.533	0.88	0.533	0.893	0.893	0.893	0.866	0.866	ID
**M**	Nucleotide (n=666)										Amino acid (n=222)									
	HQ728 482	HQ728 483	MG693 169	MG693 170	MG693 171	MG693 172	NC048 212	OK067 320	OK067 321	NRB24	HQ728 482	HQ728 483	MG693 169	MG693 170	MG693 171	MG693 172	NC048 212	OK067 320	OK067 321	NRB24
HQ728482. Eidolon bat coronavirus/Kenya/KY24/2006	ID	0.645	0.947	0.656	0.981	0.936	0.936	0.876	0.869	0.518	ID	0.65	0.977	0.663	0.99	0.963	0.963	0.9	0.895	0.873
HQ728483. Rousettus bat coronavirus/Kenya/KY06/2006	0.645	ID	0.638	0.948	0.641	0.644	0.644	0.644	0.647	0.406	0.65	ID	0.645	0.95	0.645	0.65	0.65	0.65	0.654	0.632
MG693169. Bat coronavirus CMR900	0.947	0.638	ID	0.641	0.941	0.935	0.935	0.885	0.876	0.515	0.977	0.645	ID	0.659	0.968	0.963	0.963	0.895	0.9	0.869
MG693170. Bat coronavirus CMR66	0.656	0.948	0.641	ID	0.648	0.647	0.647	0.645	0.651	0.408	0.663	0.95	0.659	ID	0.659	0.663	0.663	0.663	0.668	0.645
MG693171. Bat coronavirus CMR891-892	0.981	0.641	0.941	0.648	ID	0.933	0.933	0.878	0.872	0.519	0.99	0.645	0.968	0.659	ID	0.954	0.954	0.9	0.895	0.873
MG693172. Bat coronavirus CMR705-P13	0.936	0.644	0.935	0.647	0.933	ID	1	0.906	0.896	0.516	0.963	0.65	0.963	0.663	0.954	ID	1	0.923	0.923	0.86
NC048212. Bat coronavirus CMR704-P12	0.936	0.644	0.935	0.647	0.933	1	ID	0.906	0.896	0.516	0.963	0.65	0.963	0.663	0.954	1	ID	0.923	0.923	0.86
OK067320. Rousettus madagascariensis nobecovirus MIZ178	0.876	0.644	0.885	0.645	0.878	0.906	0.906	ID	0.975	0.501	0.9	0.65	0.895	0.663	0.9	0.923	0.923	ID	0.981	0.819
OK067321. Rousettus madagascariensis nobecovirus MIZ240	0.869	0.647	0.876	0.651	0.872	0.896	0.896	0.975	ID	0.496	0.895	0.654	0.9	0.668	0.895	0.923	0.923	0.981	ID	0.819
NRB24	0.518	0.406	0.515	0.408	0.519	0.516	0.516	0.501	0.496	ID	0.873	0.632	0.869	0.645	0.873	0.86	0.86	0.819	0.819	ID
**N**	Nucleotide (n=1424)										Amino acid (n=476)									
	HQ728 482	HQ728 483	MG693 169	MG693 170	MG693 171	MG693 172	NC048 212	OK067 320	OK067 321	NRB24	HQ728 482	HQ728 483	MG693 169	MG693 170	MG693 171	MG693 172	NC048 212	OK067 320	OK067 321	NRB24
HQ728482. Eidolon bat coronavirus/Kenya/KY24/2006	ID	0.63	0.868	0.631	0.99	0.903	0.903	0.867	0.871	0.588	ID	0.618	0.908	0.624	0.993	0.94	0.94	0.899	0.905	0.835
HQ728483. Rousettus bat coronavirus/Kenya/KY06/2006	0.63	ID	0.613	0.952	0.634	0.617	0.617	0.62	0.623	0.463	0.618	ID	0.62	0.963	0.622	0.623	0.623	0.626	0.624	0.63
MG693169. Bat coronavirus CMR900	0.868	0.613	ID	0.618	0.867	0.871	0.871	0.882	0.886	0.582	0.908	0.62	ID	0.62	0.912	0.927	0.927	0.931	0.927	0.836
MG693170. Bat coronavirus CMR66	0.631	0.952	0.618	ID	0.632	0.615	0.615	0.619	0.622	0.459	0.624	0.963	0.62	ID	0.628	0.623	0.623	0.63	0.624	0.621
MG693171. Bat coronavirus CMR891-892	0.99	0.634	0.867	0.632	ID	0.907	0.907	0.867	0.872	0.588	0.993	0.622	0.912	0.628	ID	0.946	0.946	0.901	0.908	0.84
MG693172. Bat coronavirus CMR705-P13	0.903	0.617	0.871	0.615	0.907	ID	1	0.838	0.842	0.593	0.94	0.623	0.927	0.623	0.946	ID	1	0.885	0.891	0.829
NC048212. Bat coronavirus CMR704-P12	0.903	0.617	0.871	0.615	0.907	1	ID	0.838	0.842	0.593	0.94	0.623	0.927	0.623	0.946	1	ID	0.885	0.891	0.829
OK067320. Rousettus madagascariensis nobecovirus MIZ178	0.867	0.62	0.882	0.619	0.867	0.838	0.838	ID	0.964	0.592	0.899	0.626	0.931	0.63	0.901	0.885	0.885	ID	0.965	0.855
OK067321. Rousettus madagascariensis nobecovirus MIZ240	0.871	0.623	0.886	0.622	0.872	0.842	0.842	0.964	ID	0.587	0.905	0.624	0.927	0.624	0.908	0.891	0.891	0.965	ID	0.851
NRB24	0.588	0.463	0.582	0.459	0.588	0.593	0.593	0.592	0.587	ID	0.835	0.63	0.836	0.621	0.84	0.829	0.829	0.855	0.851	ID

Table S2: Conserved domains identified in NRB24 partial ORF1a putative amino acid sequence (*n*=2365)

Location	Viral protein	Accession	Function/Property
2-137	Nsp3	cl41743	Replication/transcription complex component<br>Nobecovirus specific marker
206-730		cl40456	C-terminal transmembrane and Y domains
744-1120	Nsp4	cl40588	Replication-transcription complex assembly<br>Transmembrane domain
1132-1222		cl24800	C-terminal domain
1229-1525	Nsp5	cd21666	Main (3C-like) protease
1532-1816	Nsp6	cl40477	Vesicle formation, transmembrane-containing protein
1817-1899	Nsp7	cd21827	Replication enzyme co-factor
1903-2099	Nsp8	cd21831	Replication enzyme co-factor
2100-2211	Nsp9	cd21898	Single-stranded RNA-binding protein
2212-2342	Nsp10	cd21901	Replication enzyme co-factor
